# Copy-when-uncertain: bumblebees rely on social information when rewards are highly variable

**DOI:** 10.1098/rsbl.2016.0188

**Published:** 2016-06

**Authors:** Marco Smolla, Sylvain Alem, Lars Chittka, Susanne Shultz

**Affiliations:** 1 Faculty of Life Sciences, University of Manchester, Manchester, UK; 2 Department of Biological and Experimental Psychology, School of Biological and Chemical Sciences, Queen Mary University of London, London, UK

**Keywords:** social learning, resource distribution, social information, bumblebees, foraging, social cue

## Abstract

To understand the relative benefits of social and personal information use in foraging decisions, we developed an agent-based model of social learning that predicts social information should be more adaptive where resources are highly variable and personal information where resources vary little. We tested our predictions with bumblebees and found that foragers relied more on social information when resources were variable than when they were not. We then investigated whether socially salient cues are used preferentially over non-social ones in variable environments. Although bees clearly used social cues in highly variable environments, under the same conditions they did not use non-social cues. These results suggest that bumblebees use a ‘copy-when-uncertain’ strategy.

## 1. Introduction

Animals gain information about their environment through personal exploration (i.e. *personal information*) or by observing others (i.e. *social information*), which can generate a trade-off between costly but accurate, and cheap but potentially unreliable information [1]. Theoretical models predict that using social information indiscriminately is not adaptive; instead, animals should use social information strategically, in a context-dependent manner [2,3]. Social learning strategies, i.e. *when* and *whom* to copy, have been described [4,5], but more research evaluating strategies predicted by theoretical models is desirable [5]. Here, we predict social information use in foraging bumblebees using an agent-based model and then test how bumblebees use social information when making foraging decisions.

The selective use of social information can be explained by patterns of resource distribution, i.e. social information is more beneficial where resources are highly variable between resource patches and stable over time [6], but personal information should be favoured where there is low variance. Using social information and joining other individuals can be adaptive where resource distributions are skewed, even if individuals have to share resources, because large resource patches can provide resources far beyond the environmental average [6]. In this scenario, the value of social information would be high. Consequently, foragers should rely more on social information where resources are variable even if social information leads to exploitative competition with other individuals in the patch [7]. Experimental studies have rarely addressed the effect of resource distribution and competition, but instead focused on whether foragers choose alternative flower types given prior experience of certain cues or resource distributions [8–11].

Bumblebees use flower traits, but also the presence of conspecifics [12], to distinguish rewarding from non-rewarding flowers in an environment with heterogeneously distributed flower patches [13] and rapid changes in reward levels [14], which makes them well suited for studying the interplay of information use and resource distribution. The presence of *demonstrators* can increase the preference for novel or initially unattractive flower types in an observer [8–11]. While this can increase the success of naive bumblebees [15], experienced foragers often avoid conspecifics [11]. This is thought to reduce the probability of encountering recently exploited, empty flowers.

Here, we use an agent-based foraging model to predict information use for different resource distributions [16]. In the model, reliability of prior information about resource patches can be compromised owing to other foragers in the same patch. We tested predictions from the model with bumblebee (*Bombus terrestris*) foragers in a foraging scenario where we provided additional cues on flowers. We reduced the cue reliability by using cues that sometimes predict rewards but not always, as one might expect in the presence of other foragers. Additionally, we test whether cue-use is affected by cue type. Although in many cases individual and social learning use the same cognitive mechanisms [17], there might be sensory filters, which mediate differential efficacy of social and non-social cues [8,18]. It is therefore possible that foragers more readily attend to socially salient cues. However, if only cue reliability is important, then social and non-social cues should be equally efficient.

## 2. Material and methods

### (a) Simulating information use

**Figure 1.**
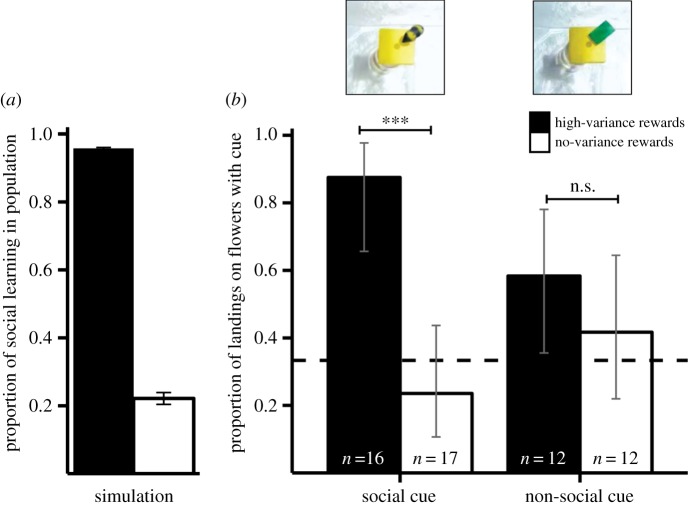
Simulations predict strong reliance on social information where rewards are highly variable, and low levels where rewards do not vary (*a*). When bees were trained and tested with bee models (social cues), we observed significantly more bees landing on a flower with a cue when they previously experienced high-variance distributions compared with the no-variance distributions (*b*). There was no difference between individuals trained and tested with the non-social cues. Dashed line indicates random choice (at 33.3%, as there were four out of 12 flowers with a cue). Error bars indicate standard errors (*a*) and 90% confidence intervals (*b*, adjusted Wald interval). ***, *p* < 0.001; n.s., non-significant.

To estimate the effect of resource distributions on social learning, we used agent-based stochastic simulations (see electronic supplementary material, S1 and [6]). We modelled foragers in a world where resources are spread among patches. Resources do not vary between patches (no-variance worlds) or vary highly between patches and change over time (high-variance worlds). Individual learners obtain information about the profitability of patches by direct exploration, whereas social learners observe other individuals exploiting patches. Individuals exploiting the same patch share resources equally through exploitative competition. The proportion of social learners evolves, as individuals die with a constant rate and are replaced by copies of the most successful foragers. We repeated simulations for each resource variability 100 times and averaged the evolved proportions of social learners in the population (figure 1*a*).

### (b) Empirical tests of information use

To test whether bumblebees conform to the model predictions and rely more on additional cues when resources are more variable, we trained bumblebees (*B. terrestris*) to forage in a flight arena. Additionally (and beyond the simulation results), we investigated whether foragers attend differently to socially salient or non-social cues. To test this, we used bee models (clay models mimicking bees in size, shape and coloration; figure 1*b*) as a proxy for a social cue and pieces of green rubber foam (15 × 8 mm) as a non-social cue. The non-social cue was selected to be maximally different from the social cue (green, flat, rectangular), but still recognizable by the bees. Earlier studies have successfully used objects as diverse as coins, wooden blocks and plastic discs as non-social cues in bumblebees [8,19].

### (c) Experimental set-up

We tested bees in a flight arena (electronic supplementary material, S2), which contained 12 artificial flowers in a regular 3 × 4 array. Foraging bouts were limited to 5 min, and each forager completed five bouts per training phase. Training and tests were conducted on the same day.

### (d) Training phase one: reliability of cues indicating food

Individual foragers were trained to find rewarding flowers. Four out of 12 transparent flowers had either social or non-social cues attached (figure 1*b*). To alter cue reliability and mimic resource depletion of rewarding flowers two of the flowers with an additional cue were empty and two were filled with 30 µl 30% sucrose solution each. Flowers without additional cues were water-filled (30 µl).

### (e) Training phase two: different reward variance in flower arrays

Subsequently, bees encountered one of two resource distributions in a novel training world containing yellow flowers, without additional cues. 100 µl of 30% sucrose solution either was equally divided among all 12 flowers (*no-variance*) or was divided between only two, the rest being filled with water (50 µl each, *high-variance*).

### (f) Test phase: test, combining cues and flowers

In the test, the flower array consisted of 12 yellow, water-filled flowers, of which four had an additional social or non-social cue, consistent with phase one. Bees were allowed only one foraging bout. The position of flowers with cues was consistent for all individuals.

### (g) Data recording and analysis

Landings (the bee extending its proboscis into the flower cavity, or resting its wings after placing its legs on the flower) were recorded in all flights. To keep conditions similar for all individuals all reported values regard the first landing after the bee entered the foraging arena (see electronic supplementary material, S3 for additional data). The first flower choice is before bees from the no-variance treatment experience unrewarded yellow flowers, contrary to their previous training.

We used binomial tests to test whether the proportion of bees landing on flowers with an additional cue differs from chance.

We fitted a logistic regression model to determine variables that significantly predicted the first flower choice of the bee (binary: 1 = flower with cue, 0 = flower without cue). The model included cue type (bee model = 1, foam model = 0) and reward distribution (no-variance = 1, high-variance = 0) as fixed effects with an interaction term, and colony as a random effect. To explore the interaction between cue type and reward distribution more directly, we split the data by cue type, and evaluated a model with reward distribution as a fixed and colony as a random effect (figure 1*b*). Data analysis was performed using R [20] and the lme4 R-package [21].

## 3. Results

Our simulation predicted high proportions of social learners where resources are highly variable (0.96 ± 0.002, figure 1*a*), and low proportions where resources do not vary (0.22 ± 0.02). We therefore expected foragers from the high-variance treatment to prefer landing on flowers with additional cues, whereas bees from the no-variance treatment should not exhibit a preference for these flowers.

We tested 57 bees from three colonies. Only bees from the social cue, high-variance treatment landed first on flowers with cues more than would be expected by chance alone (binomial test, *n* = 16, *p* < 0.001). All other groups did not significantly differ from random choice (binomial test for all *p* > 0.05). While the interaction between cue type and reward distribution was significant, the main effects were not significant (logistic regression; *n* = 57, cue type = −1.138 ± 0.891, *z* = −1.278, *p* = 0.201, reward distribution = 0.733 ± 0.867, *z* = 0.846, *p* = 0.398, cue type : reward distribution = 2.593 ± 1.309, *z* = 1.981, *p* = 0.048). When social cue was analysed separately, bees from the high-variance treatment were more likely to land on flowers with cues than bees from the no-variance treatment (logistic regression; *n* = 33, reward distribution = 3.125 ± 0.948, *z* = 3.297, *p* < 0.001, figure 1*b*), which is in qualitative agreement with our simulation. However, cue-use in bees from the non-social cue training did not show a difference based on prior experienced resource variability (logistic regression; *n* = 24, reward distribution = 0.696 ± 0.846, *z* = 0.823, *p* = 0.411). The overall pattern persists even when averaging landings over the first four and 10 landings (see electronic supplementary material, S3). The difference in using social and non-social cues, however, cannot be attributed to different learning success throughout the cue training as bees had landed on both cue types more than would be expected by chance alone by the end of the first training independent of the resource distribution they were trained with (binomial test for all *p* < 0.001, data not shown).

## 4. Discussion

Based on simulations, we expected experimental bumblebees to use provided cues when they had experienced highly variable rewards. Bees from the high-variance treatment indeed preferentially landed on flowers with social cue proxies (bee models). Furthermore, cue-use only differed between reward distributions when bees were trained to social but not to non-social cues. This shows that bumblebees responded to high exploration costs by using available social information. It was recently found that bumblebees do not prioritize social information over previous personal experience [22], and therefore did not display a ‘copy-when-established-behaviour-is-unproductive’ strategy [5]. Our results suggest that bumblebee foragers use a ‘copy-when-uncertain’ strategy instead.

Although our computational model did not allow individuals to switch between individual and social learning (which in itself offers nearly unbounded possible implementations, see [2]), we obtained intuitive results showing that acquiring information socially can be adaptive where rewards are clumped and difficult to locate [23]. Honeybees, for example, attend waggle dances more fully when artificial feeders are further away from the hive [24]. When resources are easily shareable, such as with flowering trees and other single large rewards, competition is reduced, and the value of socially acquired information is thus increased [23]. Following an individual to a single large resource therefore reliably predicts a reward. However, social information has little value when following another forager to a small and easily depleted resource, where competition is strong. Here, the presence of another individual would not reliably predict a reward.

In our experiments, we mimicked exploitative competition by reduced cue reliability, because as with live demonstrators, with which an observer bee would compete for resources, their presence would not always reliably predict a reward. Owing to the highly skewed resource distribution in the high-variance treatment, foragers would be three times more likely to find a reward on a flower with a cue than flowers without a cue. Previous work showed that the use of available social information was only adaptive when naive bumblebees foraged in an environment where rewards were patchily distributed [15]. Our results suggest that this is equally true for experienced foragers.

While non-social cues were as much used as social cues at the end of the cue training, bees neither preferred nor avoided them in the test, showing that bumblebees do not use cues based on reliability and reward distribution alone but also according to their nature. This is consistent with earlier studies revealing that socially salient cues are most efficient in learning tasks (see also [8–10]), whereas other non-social cues are used less [17,19,25]. Conspecifics might have indicated rewarding flowers reliably enough to induce the evolution of attentional, perceptual or motivational mechanisms biased towards social cues [17]. Our study supports not only the view that social learning strategies exist in relatively simple organisms [26], but also that mechanisms exist which allow treating social and non-social cues differently.

## Supplementary Material

Supplementary information

## Data accessibility

Dryad: http://dx.doi.org/10.5061/dryad.3jb68.
