# Supplementary material for: Copy-when-uncertain: bumblebees rely on social information when rewards are highly variable
Source: Biol Lett. 2016 Jun;12(6):20160188. doi: 10.1098/rsbl.2016.0188 (PMC4938046; doi:10.1098/rsbl.2016.0188)
Supplement: Supplementary information [file rsbl20160188supp1.pdf]

Electronic supplementary material

Copy-when-uncertain: bumblebees rely on social information when rewards are highly variable

Marco Smolla[1,2], Sylvain Alem[2], Lars Chittka[2], Susanne Shultz[1]

[1]Faculty of Life Sciences, University of Manchester, Manchester, UK

[2]Department of Biological and Experimental Psychology, School of Biological and Chemical Sciences, Queen Mary University London, London, UK

S1 Social learning model

The model we use here is adapted from the model described by [1]. As with the previous model the environment consisted of patches and each patch provided an amount of π resources. However, we changed the average resource value from four to 8.3 in all simulations to make the model comparable with the experimental foraging arena; where there were 12 flowers (similar to [2]), and an average patch size of 100μl (based on the body-size dependent crop volume of bumblebees ranging from 75–180μl [3,4]). Resources either did not vary between patches (*no-variance* distribution, all $\pi = 8.3$) or varied strongly (*high-variance*, π drawn from a gamma distribution with $k = 0.183$ and $\theta = 45.45$; for comparison with our earlier model [1] this equates to a Gini index of $G = 0.81$). The average amount of resources was the same in both conditions. In high-variance simulations patch resources π change with a constant probability $\tau = 10^{-4}$ in each time-step. In every time-step all patches are replenished to their according value of π.

Individuals forage for resources in patches. Individuals can exploit patches they know about, either through personal exploration (*individual learning*) or through observation of other individuals exploiting a patch (*social learning*). Each individual is either an individual learners or a social learner. Individual learners learn about a randomly selected new patch, while social learners learn about a patch of a randomly selected individual that exploited a patch in the last round. In both cases individuals learn the amount of resources a patch currently provides, i.e. its *anticipated reward*. Individuals exploit with probability $\beta = 0.8$ (thus, they learn with probability $1-\beta$). If

an individual chooses to exploit, it selects the patch with the highest anticipated reward from its memory. The amount of resources the individual collects is $p = \pi/n$, where n is the number of individuals that chose the same patch in this time-step. Thus, individuals engage in exploitative competition and equally share resources.

Information about anticipated rewards persists in an individual's memory until it is updated either when the individual learns the same patch again or gains resources different from the anticipated reward, or until the individual dies.

Individuals die with a probability of $d = 0.02$ in each time-step, or when they exceed 100 time-steps. When an individual dies, it is replaced with a new individual with no knowledge of the environment (i.e. empty memory). Thus, the population size is constant. The proportion of social learners evolves: individuals die at a constant rate and are replaced by individuals with the learning strategy of one of the surviving individuals, probabilistically selected relative to their fitness. For example, assuming there are two surviving individuals, a social learner with fitness 0.5 and an individual learner with fitness 2, then the new individual will be an individual learner with a 75% chance. We use the relative amount of resources collected by an individual as a performance measure that is commonly used as fitness proxy in foraging theory [5]. This way, the initial proportion of individual and social learners (1:1) evolves to an equilibrium point where the fitness of individual and social learners does not differ.

We ran simulations with 100 patches and 33 individuals for 10,000 time-steps. In each of the last 2,500 rounds of each simulation, we recorded the proportion of individual and social learners. We conducted 100 simulations for each resource variance. Resource variance (high/no-variance) and the ratio between cues and flowers (1:3) of our empirical experiments matched those of our simulation.

S2 Animals and treatment

We used three bumblebee colonies (*Bombus terrestris*), provided by Koppert (Netherlands). The colony was housed in a wooden box (270 x 400 mm and 110 mm high) connected to an additional wooden box (120 x 140 mm and 110 mm high), which we used as feeding arena, where we provided 20% sucrose solution before and

after experiments (approx. 75 ml per day). This ensured low colony reserves and a high motivation to forage. Pollen was provided directly onto the nest every other day. The colony was also connected to a flight arena (700 x 510 mm; 300 mm high, covered by UV-transparent Plexiglas) with a Plexiglas tunnel (250 mm length; 35 x 35 mm cross-section) fitted with sliding doors to control the traffic of bees. The flight arena contained twelve artificial flowers (10 x 10 mm plastic chips with a central cavity 2 mm in diameter, placed on top of 40 mm high glass vials) in a regular 3 x 4 array (distance between flowers 80 mm). Flowers were cleaned with ethanol wipes and refilled between foraging bouts. In the training sessions, the position of rewarding flowers was randomized between foraging bouts. Light conditions in the flight arena mimicked the natural daylight spectrum and the light flicker frequency was set beyond bumblebees' flicker fusion frequency [6]. Illumination followed a 12h/12h photoperiod. Individual bees were marked with numbered tags (Opalithplättchen, Warnholz & Bienenvoigt, Germany) or paint marks to allow identification, and removed from the colony after testing. Training and testing was conducted between 8:00AM and 7:00PM.

S3 Additional observations beyond the first landing

For all test flights we observed all landings on flowers. In Fig. S1*a* and S2*a,* additional to the proportion of bees landing on flowers with a cue ('first', which is identical to the results in Fig. 1*b*), we report the average proportion of landings on flowers with social or non-social cues for the 'first four' and 'first ten' landings. Again for bees trained with social cues we find significant differences between bees from the high- and no-variance treatment (Pearson's Chi-squared test for first four landings: $P < 0.01$; Pearson's Chi-squared test for first four landings: $P < 0.001$, Fig. S1*a*). However, bees trained with non-social cues did not differ from each other, neither for the first four nor for the first ten landings (Pearson's Chi-squared test for both: $P > 0.05$, Fig. S2*a*). Only bees trained to social cues and the high-variance treatment landed more often flowers with a social cue in their first four and first ten landings than would be expected by chance alone (binomial test for both: $N = 16$, $P < 0.001$, Fig. S1*a*). Bees from all the other treatments did not land more on flowers with a cue than would be expected by chance alone (binomial test for social cue, no-variance, and non-social cue and high- and no-variance: $P > 0.05$, Fig. S1*a* and S2*a*).

In Fig. 1*b* of the main text we present only results for the first landing while earlier studies have shown that the decision to use social information can strongly differ between first and subsequent landings [7]. Based on the effect a rewarded flower without a cue or a non-rewarded flower with a cue could have on the subsequent choice, we decided to only look at the first landing because at this stage all foragers have the same knowledge about yellow flowers and cues. Especially for bees from the no-variance treatment this is the first encounter of yellow, water-filled.

The results for the subsequent landings show a slight increase (no-variance, Fisher's Exact test $P = 0.16$) or slightly decreased (high-variance treatment, Fisher's Exact test $P = 0.39$) in the use of cues for bees from the social cue training between the first and the second landing (Fig. S1*b*). Interestingly, for bees from the non-social cue training this effect appears to be reversed between the two different treatments, where the use of non-social cues was slightly decreased (high-variance, Fisher's Exact test $P = 0.09$) or showed no change (no-variance, Fisher's Exact test $P = 1$) (Fig. S2*b*). However, none of these are statistically significant.

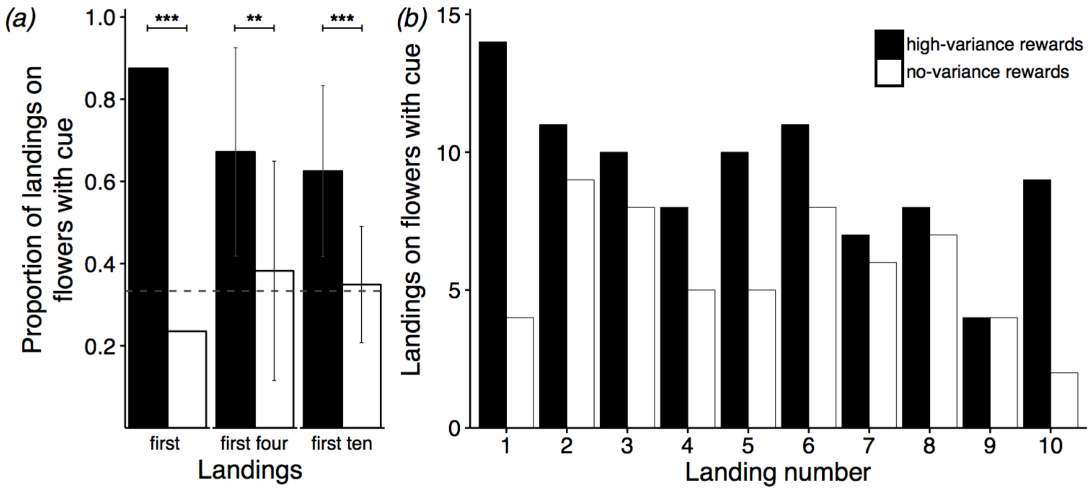

Figure S1: Bees trained in the high variance, social cue treatment landed on flowers with social cues more often than would be expected by chance alone. This effect persists throughout the first four and first ten landings (*a*). Also, at the second landing slightly more bees from the no-variance treatment land on flowers with social cues (*b*). Error bars represent SD; dashed line represents random choice; asterisks represent level of statistical significance ('**': P=0.01, '***': P<0.001).

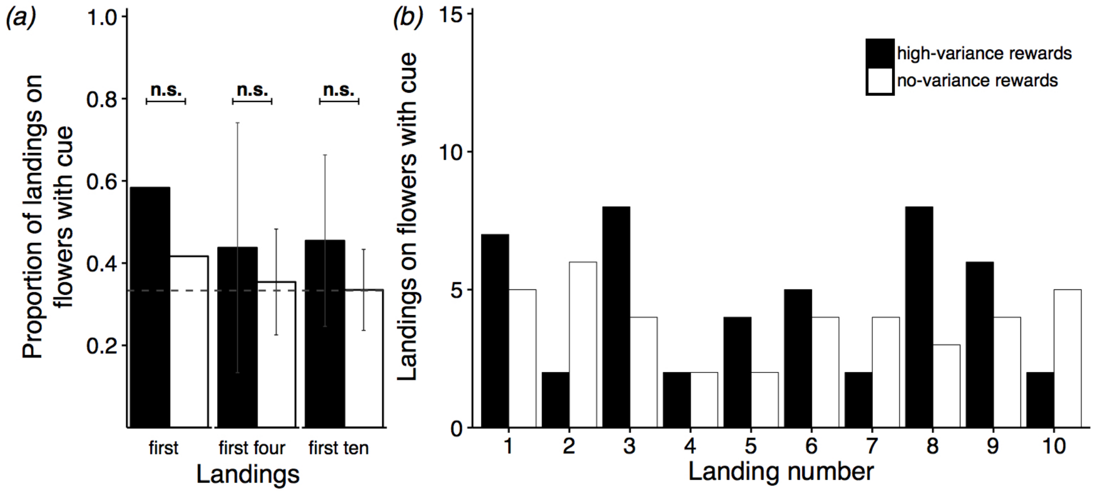

Figure S2: Bees trained to non-social cues did not differ in their preference for landing on flowers with cues and also did not land more often on flowers with cues than would be expected by chance alone (*a*). Also, at the second landing slightly less bees from the high-variance treatment land on flowers with non-social cues (*b*). Error bars represent SD; dashed line represents random choice.

References

1. Smolla, M., Gilman, R. T., Galla, T. & Shultz, S. 2015 Competition for resources can explain patterns of social and individual learning in nature. *Proc. R. Soc. B Biol. Sci.* **282**, 20151405. (doi:10.1098/rspb.2015.1405)
2. Avarguès-Weber, A. & Chittka, L. 2014 Local enhancement or stimulus enhancement? Bumblebee social learning results in a specific pattern of flower preference. *Anim. Behav.* **97**, 185–191. (doi:10.1016/j.anbehav.2014.09.020)
3. Lihoreau, M., Chittka, L., Le Comber, S. C. & Raine, N. E. 2012 Bees do not use nearest-neighbour rules for optimization of multi-location routes. *Biol. Lett.* **8**, 13–6. (doi:10.1098/rsbl.2011.0661)
4. Lihoreau, M., Chittka, L. & Raine, N. E. 2010 Travel optimization by foraging bumblebees through readjustments of traplines after discovery of new feeding locations. *Am. Nat.* **176**, 744–757. (doi:10.1086/657042)

5. Rendell, L. et al. 2010 Why copy others? Insights from the social learning strategies tournament. *Science* **328**, 208–13. (doi:10.1126/science.1184719)

6. Skorupski, P. & Chittka, L. 2010 Differences in photoreceptor processing speed for chromatic and achromatic vision in the bumblebee, *Bombus terrestris*. *J. Neurosci.* **30**, 3896–3903. (doi:10.1523/JNEUROSCI.5700-09.2010)

7. Baude, M., Danchin, E., Mugabo, M. & Dajoz, I. 2011 Conspecifics as informers and competitors: an experimental study in foraging bumble-bees. *Proc. Biol. Sci.* **278**, 2806–2813. (doi:10.1098/rspb.2010.2659)
